# Interaction of Docetaxel with Phosphatidylcholine Membranes: A Combined Experimental and Computational Study

**DOI:** 10.1007/s00232-022-00219-z

**Published:** 2022-02-17

**Authors:** Elisa Aranda, José A. Teruel, Antonio Ortiz, María Dolores Pérez-Cárceles, Francisco J. Aranda

**Affiliations:** 1 Departamento de Bioquímica y Biología Molecular-A, Facultad de Veterinaria, Universidad de Murcia, 30100 Murcia, Spain; 2 Departamento de Medicina Legal y Forense, Facultad de Medicina, Instituto de Investigación Biomédica (IMIB-Arrixaca), Universidad de Murcia, 30120 Murcia, Spain; 3 Present Address: Hospital Universitario Virgen de la Arrixaca, Área de Salud 1, Murcia, Spain

**Keywords:** Docetaxel, DPPC, DSC, X-ray diffraction, FTIR, Molecular dynamics

## Abstract

The antineoplastic drug Docetaxel is a second generation taxane which is used against a great variety of cancers. The drug is highly lipophilic and produces a great array of severe toxic effects that limit its therapeutic effectiveness. The study of the interaction between Docetaxel and membranes is very scarce, however, it is required in order to get clues in relation with its function, mechanism of toxicity and possibilities of new formulations. Using phosphatidylcholine biomimetic membranes, we examine the interaction of Docetaxel with the phospholipid bilayer combining an experimental study, employing a series of biophysical techniques like Differential Scanning Calorimetry, X-Ray Diffraction and Infrared Spectroscopy, and a Molecular Dynamics simulation. Our experimental results indicated that Docetaxel incorporated into DPPC bilayer perturbing the gel to liquid crystalline phase transition and giving rise to immiscibility when the amount of the drug is increased. The drug promotes the gel ripple phase, increasing the bilayer thickness in the fluid phase, and is also able to alter the hydrogen-bonding interactions in the interfacial region of the bilayer producing a dehydration effect. The results from computational simulation agree with the experimental ones and located the Docetaxel molecule forming small clusters in the region of the carbon 8 of the acyl chain palisade overlapping with the carbonyl region of the phospholipid. Our results support the idea that the anticancer drug is embedded into the phospholipid bilayer to a limited amount and produces structural perturbations which might affect the function of the membrane.

**Graphical Abstract:**

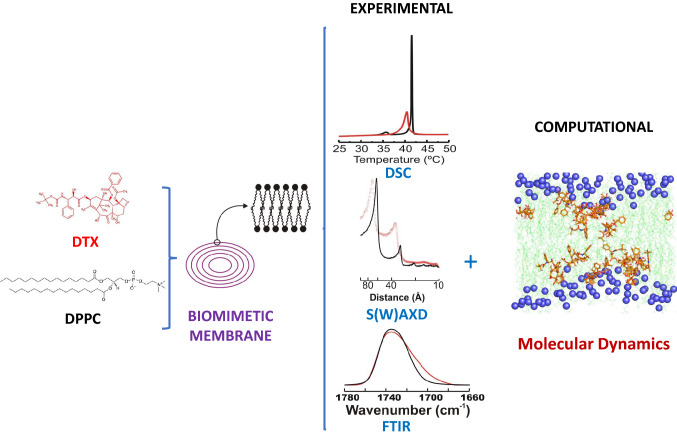

## Introduction

**Fig. 1:**
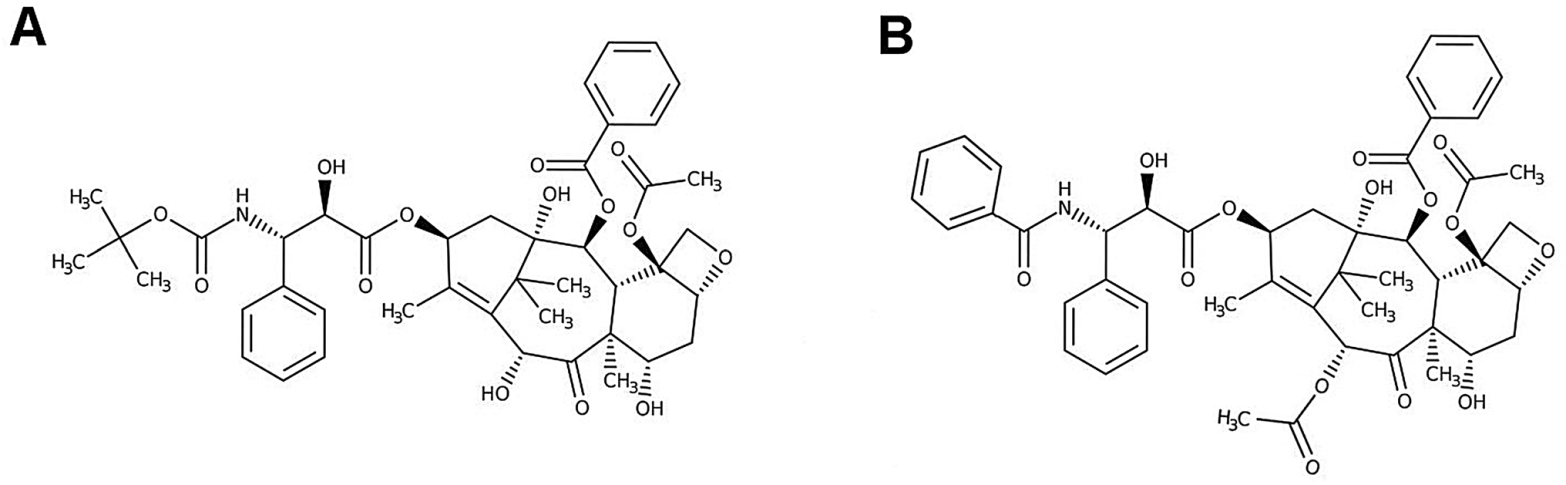
Chemical structure of **A** Docetaxel and **B** Paclitaxel

Docetaxel is an anticancer drug which is a member of the second generation of taxanes. The taxanes family embodies Paclitaxel and Docetaxel, Paclitaxel being a diterpenoid natural product extracted from the bark of the Pacific yew (*Taxus brevifolia*) (Wani et al 1971). Docetaxel (DTX, Fig. 1) is a semi-synthetic analogue of Paclitaxel, prepared from precursor extracted from the needles of *Taxus baccata*, 10-deacetyl baccatin III (Bissery and Gueritte-Voegelein 1991). DTX differs from Paclitaxel in two positions, it has a hydroxy functionality at C-10 instead of the acetate ester found in Paclitaxel and the bulky phenylpropionate side chain has a tert-butyl substitution attached by means of a carbamate linkage (Clarke and Rivory 1999).

Taxanes are used in the remedy of a wide spectrum of cancers, including refractory ovarian (Adler et al. 1994; Katsumata 2003), breast (Jones et al. 2005; Lyseng-Williamson and Fenton 2005), non-small cell lung (Quiox et al. 2004; He et al. 2015), gastric (Uson Junior et al. 2019) and prostate cancer (Assi et al. 2020). The principal mechanism of action of taxanes underlies in their microtubule stabilizing properties which prevent physiological microtubule depolymerization and disassembly, leading to cell cycle arrest at the G2/M phase and cell death (Tan et al. 2012). Antineoplastic drugs must cross the plasma membrane to elicit pharmacological activity (Bourgaux and Couvreur 2014), consequently, through their biological route the interaction with the membrane becomes unavoidable. The interaction of DTX with the membrane is a complex physical and chemical event, which can affect the rate of penetration of the drug into the cytoplasm where it must reach its specific target. Hence, the interaction of DTX with the membrane should be studied in order to get insight into its mechanism of action.

DTX has been described to originate severe toxic side effects which include neutropenia (de Vries Schultink et al. 2019), musculo skeletal toxicity (Seguin et al. 2017), peripheral neuropathy (Cheng et al. 2021), hypersensitivity reactions (Picard and Castells 2015) and skin toxicity (Arwert 2009). A series of clinical studies have revealed that DTX is more toxic than Paclitaxel. DTX induced the presence of a unique fluid retention syndrome (Rowinsky 1997), it caused more neutropenia, thrombocytopenia, and onycholysis in axillary node-positive early breast cancer patients (Saloustros et al. 2014), shows a tendency towards higher non-haematological toxicities in patients with non-small-cell lung cancer (Esteban et al. 2003), and caused more frequent hematologic and nonhematologic toxicities in metastatic breast cancer patients (Jones et al. 2005). In this regard, DTX-lipids interactions may modify the structure of the membrane, alter its function and also contribute to the mechanism of DTX toxic effects.

In this context, the interaction between taxanes and membrane, and its consequence for its structure and properties, constitutes a considerable field of study. A series of studies have used bilayer model membranes to study the interaction between Paclitaxel and dipalmitoylphosphatidylcholine (DPPC) (Balasubramanian and Straubinger 1994; Zhao et al. 2004), different saturated and unsaturated phosphatidylcholines (Bernsdorff et al. 1999; Zhao and Feng 2004, 2005), using bromoacylated taxanes (Ali et al. 2000), pegylated lipids (Belsito et al. 2005) and in the presence of cholesterol (Zhao et al. 2007). DTX has attained enormous consideration in cancer chemotherapy as a result of its good therapeutic index, exhibiting enhanced efficacy when compared with Paclitaxel (Imran et al. 2020). DTX is more water soluble and promptly absorbed in relation to paclitaxel due to the variation in their chemical structure, showing considerable differences in cytotoxic and antineoplastic actions (da Silva et al. 2018). Despite all the above, studies on the interaction between DTX and phospholipids are very scarce. The interaction between DTX and DPPC has been studied using lipid monolayers at the air–water interface (Fernández-Botello et al. 2008), and the interaction between DTX and dimyristoylphosphatidylcholine has been studied by Differential Scanning Calorimetry (Sarpietro et al. 2013). For the best of our knowledge, the comprehensive study of the interactions between DTX and DPPC bilayers has not been previously carried out.

In this work, we present a combined approach to study DTX interactions with DPPC bilayers, using diverse complementary biophysical techniques, specifically high sensitivity Differential Scanning Calorimetry (DSC), Small and Wide X-ray Diffraction (SAXD and WAXD) and Fourier Transform Infrared Spectroscopy (FTIR), as well as Molecular Dynamics (MD). The central aim of this study is to thoroughly examine and characterize the molecular interactions between DTX and DPPC bilayers in order to obtain information that we believe might contribute to a better understanding of the process of internalization and accumulation inside the target cell affecting the therapeutic efficacy and the mechanism of toxicity of the drug.

## Materials and Methods

### Materials

1,2-Dipalmitoyl-sn-glycero-3-phosphocholine (Dipalmitoylphosphatidylcholine, DPPC, > 99% TLC) was obtained from Avanti Polar Lipids Inc. (Birmingham, AL). Phospholipid concentration was determined by phosphorous analysis (Böttcher et al. 1961). Docetaxel [4-acetoxy-2α-benzoyloxy-5β,20-epoxy-1,7β,10β-trihydroxy-9-oxotax-11-ene13α-yl-(2R,3S)-3-tert-butoxycarbonyl-amino-2-hydroxyphenylpropionate] (DTX, ≥ 98.0%) was obtained from Glentham Life Sciences (UK). Purified water was deionised in a Milli-Q equipment from Millipore (Bedford, MA), and filtered through 0.24 μm filters prior to use. All other reagents were of the highest purity available.

### Differential Scanning Calorimetry

The lipid mixtures for DSC measurements were prepared by combination of chloroform solutions containing DPPC and the appropriate amount of DTX as indicated. The organic solvent was evaporated under a stream of dry N_2_, free of O_2_, and the last traces of solvent was removed by further 3 h evaporation under high vacuum. To the dry samples, 0.5 ml of a buffer containing 150 mM NaCl, 0.1 mM EDTA, 10 mM Hepes pH 7.4 was added, and biomimetic multilamellar vesicles were formed by vortexing the mixture, at temperature above the gel to liquid-crystalline phase transition temperature of the phospholipid. Experiments were performed using a MicroCal DSC PEAK calorimeter (Malvern Panalytical). The final phospholipid concentration was 1.5 mM, and the heating scan rate was 60 °C h^−1^. Three consecutive heating scans were carried out for each sample. Thermograms from the second and third scan were identical, the last one being taken for analysis. The integral of heat capacity over temperature gives the calorimetric enthalpy for the transition. Peak areas under the thermograms relative to the baseline was determined as a direct measurement of the enthalpy of the transition. Data were analyzed using ORIGIN software provided by MicroCal. The construction of partial phase diagrams was based on the heating thermograms for a given mixture of phospholipid and drug at various drug concentrations. The onset and completion temperatures for each transition peak were obtained from the heating thermograms taken at the points of intersection of the tangents to the leading edges of the endotherms and the baselines, and were plotted as a function of the molar fraction of drug. These onset and completion temperatures points formed the basis for defining the boundary lines of the partial temperature-composition phase diagram.

### X-ray Diffraction

Simultaneous small (SAX) and wide (WAX) angle X-ray diffraction measurements were carried out using a modified Kratky compact camera (MBraum-Graz-Optical Systems, Graz Austria) which employs two coupled linear position sensitive detectors (PSD, MBraum, Garching, Germany). Nickel-filtered Cu K_α_ X-rays were generated by a Philips PW3830 X-ray Generator operating at 50 kV and 30 mA. Samples for X-ray diffraction were prepared by mixing 10 μmol of DPPC and the appropriate amount of DTX in chloroform, and multilamellar vesicles were formed as described above. After centrifugation at 13,000 rpm, the pellets were placed in a steel holder, which provided good thermal contact to the Peltier heating unit, with cellophane windows. Exposure times were 10 min, allowing 10 min prior to the measurement for temperature equilibration. Background corrected SAXD data were analysed using the program GAP (Global Analysis Program) written by Prof. Georg Pabst (University of Graz, Austria) and obtained from the author (Pabst et al. 2000, 2003). This program allowed to retrieve the membrane thickness, *d*_B_ = 2(*Z*_H_ + 2σ_H_) from a full q-range analysis of the SAXD patterns (Pabst 2006). The parameters Z_H_ and σ_H_ are the position and width, respectively, of the Gaussian used to describe the electron-dense headgroup regions within the electron density model. The width σ_H_ was fixed to 3 Å.

### Fourier Transform Infrared Spectroscopy (FTIR)

Samples for the infrared measurements containing 10 μmol of DPPC and the appropriate amount of DTX were formed in 75 μl of the same buffer prepared in D_2_O as described above. Samples were placed in between two CaF_2_ windows (25 × 2 mm) separated by 25 μm Teflon spacers and transferred to a Symta cell mount. Infrared spectra were acquired in a Nicolet 6700 FTIR spectrometer (Madison, WI). Each spectrum was obtained by collecting 64 interferograms with a nominal resolution of 2 cm^−1^. The equipment was continuously purged with dry air in order to minimize the contribution peaks of atmospheric water vapor. The sample holder was thermostatized using a Peltier device (Proteus system from Nicolet). Spectra were collected at 1 °C intervals, allowing 5 min equilibration between temperatures. The D_2_O buffer spectra taken at the same temperatures were subtracted interactively using either Omnic or Grams (Galactic Industries, Salem, NH) software.

### Molecular Dynamics (MD)

The 3D molecular structure of DTX was obtained from the Cambridge Crystallographic Data Centre, CCDC No. 940082 (Cambridge Crystallographic Data Center 2021; Groom et al. 2016)⁠. All MD simulations were done using GROMACS 5.0.7 and 2018.1 (Abraham et al. 2015) in the Computational Service of the University of Murcia (Spain). CHARMM36 force field parameters for DPPC, DTX, water, Cl^−^ and Na^+^ were obtained from CHARMM-GUI (Jo et al. 2000; Brooks et al. 2009; Lee et al. 2016). The membrane bilayer was formed by 2 leaflets oriented normal to the z-axis with a total of 124 molecules of DPPC with and without 12 molecules of DTX in the lipid phase, and a water layer containing a total of 6000 water molecules (TIP3 model), 12 sodium ions, and 12 chloride ions. Three independent simulations were performed using the above molecular distribution of the system. The initial membrane structures were built with the aid of Packmol software (Martínez et al. 2009), where all molecules were randomly distributed in each layer keeping the DPPC molecules oriented normal to z-x, and thus in the different simulations the DTX molecules were at different random starting locations in the lipid phase.

All systems (DPPC and DPPC + DTX) were simulated using the NpT-ensamble at 50 °C. Pressure was controlled semi-isotropically at a pressure of 1 bar and compressibility of 4.5 × 10^−5^ bar^−1^. The cutoffs for van der Waals and short-range electrostatic interactions were 1.2 nm, and a force switch function was applied between 1.0 and 1.2 nm (Bjelkmar et al. 2010). Simulations were initiated by an equilibration run for 100 ns, using the V-rescale thermostat and the Berendsen barostat (Berendsen et al. 1984)⁠, followed by a production run of 100 ns using the Nose–Hoover thermostat (Hoover 1985) and the Parrinello-Rahman barostat (Parrinello and Rahman 1981). Graphical representations were done with PyMOL 2.3.0 (Schrödinger 2010). Analysis of the trajectories were done over the last 60 ns of the production run trajectories using the Gromacs tools.

## Results and Discussion

### Differential Scanning Calorimetry (DSC)

We used DSC in order to characterize the influence of DTX on the thermotropic properties of DPPC bilayers and found that the drug perturbed the gel to liquid crystalline phase transition of the phospholipid.

**Fig. 2:**
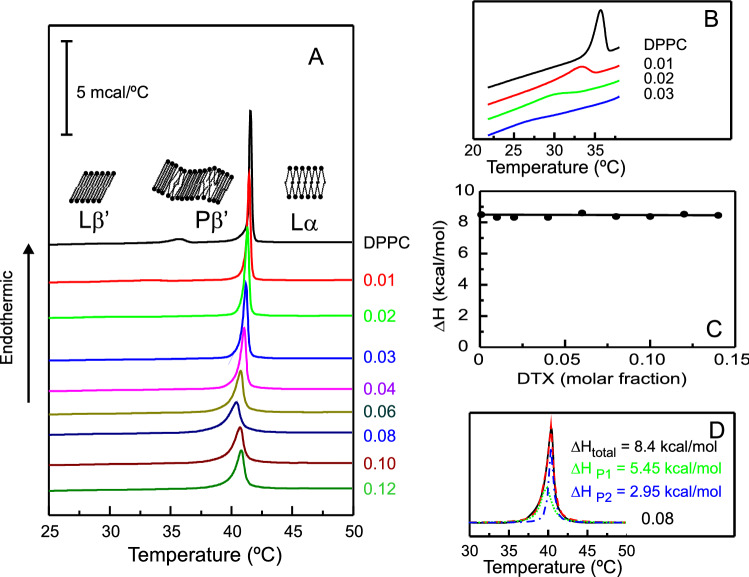
**A** DSC heating thermograms for pure DPPC and DPPC containing DTX at different concentrations. **B** Enlargement (× 16) of the pretransition region of the thermograms. **C** Enthalpy change for the main gel to liquid-crystalline phase transition of DPPC containing DTX at different concentrations. **D** Fitting of the baseline subtracted thermogram corresponding to DPPC containing DTX to two transition peaks. Original thermogram (solid line), fitted thermogram (dashed line), low temperature transition peak (dotted line) and high temperature transition peak (dashed dotted line). Enthalpy changes for the fitted peak transitions are indicated. DTX molar fraction are expressed on the right side of the thermograms

The effect of DTX on the thermotropic phase transition of DPPC is illustrated in Fig. 2. Pure DPPC (Fig. 2A, top thermogram) at low temperature is organized in the lamellar gel phase, L_β_’, where the acyl chains are in the all-trans conformation, fully extended and tilted with respect to the bilayer normal. At 35 °C, the phospholipid undergoes a low enthalpy pretransition which transforms the L_β_’ into the P_β_’ phase in which the bilayer is distorted by a periodic ripple. Around 41 °C the higher enthalpy main phase transition transforms the gel P_β_’ phase into the fluid or liquid crystalline phase, L_α_, in which gauche rotational isomerization takes place and the acyl chains are perpendicular to the bilayer plane, in agreement with previous results (Cevc and Marsh 1987; Bourgaux and Couvreur 2014).

As depicted in Fig. 2, the presence of DTX gives rise to an alteration of the thermotropic and structural parameters of DPPC. As presented in Fig. 2A, it seems that the pretransition has already disappeared in the presence of DTX at 0.01 molar fraction, this being in agreement with the report that in dimyristoylphosphatidylcholine system, the pretransition peak disappeared in the presence of DTX 0.015 molar fraction (Sarpietro et al. 2013). However, the enlargement of the 22–37 °C region presented in Fig. 2B shows that at these low concentrations of DTX the pretransition is clearly detected, though broadened and shifted to lower temperatures. Only at DTX 0.04 molar fraction the pretransition could not be detected. This difference could be due either to the high sensitivity calorimeter used in our experiments or to a more pronounced effect of DTX on the shorter homologue dimyristoylphosphatidylcholine.

The presence of increasing concentrations of DTX gradually broadens the main phase transition and produces a displacement to lower temperatures until reaching a DTX 0.08 molar fraction, from which higher concentrations no longer have any additional effect. The width of the transition is an estimation of destabilization of the phospholipids association, related to the size of the cooperative unit and being a measure of the intermolecular interactions between phospholipids molecules (Lewis et al. 2007). The presence of DTX into DPPC bilayers induced a decrease in the cooperativity of the gel to liquid crystalline phase transition. The onset of the main phase transition temperature decreases from 41.2 °C for pure DPPC till 38.9 °C in the presence of DTX 0.08 molar fraction. The changes caused on the main phase transition suggest a localization of DTX in the hydrophobic cooperative region of the bilayer. The enthalpy change of the main phase transition for pure DPPC was found to be of 8.5 kcal mol^−1^, and it was not substantially altered in the presence of DTX (Fig. 2C). Since the enthalpy change reflects the heat absorbed during the melting of all the phospholipid acyl chains, it suggests that in the presence of DTX all phospholipid molecules in the system still undergo the phase transition.

The phase transition thermograms in the presence of DTX are clearly broadened and asymmetrical and this may reflect a non-uniform DTX distribution in the bilayer. Figure 2D shows that the thermogram of the sample containing DTX 0.08 molar fraction can be fitted to two transition peaks, a broad lower melting peak encompassing 65% of the enthalpy change of the transition, and a sharp higher melting peak encompassing the remaining of the enthalpy change. This behaviour probably reflects the formation of two distinct domains in the bilayer containing different populations of DTX.

The ratios between DTX and phospholipid used in this study are similar to those commonly used in previous studies on the interaction between taxanes and membranes. The correlation between these ratios and the concentrations of DTX exhibiting anticancer activity is not direct, but an approximation can be made. It has been reported for an epithelial cell line that the phospholipid content is around 2 μg Pi/10^6^ cells (Casali et al. 2013). Assuming that the phospholipid content in other cancer cell lines is similar to that value and considering that concentration of DTX usually range between 10 and 120 nM, it renders that under a cell culture condition such as 4 × 10^4^ cells and a volume of the medium of 0.1 ml (Trebunova et al., 2012) the DTX/phospholipid ratio in physiological studies might reach a value from 0.01 to 0.10 which is very close to the values used in our study. In addition, we would like to point out that the molar ratios studied in our model system are not necessarily required to be homogeneous in the whole cellular membrane, it would be enough that this DTX/phospholipid ratio be attained locally in certain parts of the membrane.

In the following studies, a DTX concentration of 0.08 molar fraction was selected in order to study the maximal action of the drug on the properties of DPPC bilayers both below and above the main phase transition temperature.

### X-ray Diffraction

We have used Small and Wide Angle X-Ray Diffraction (SAXD and WAXD) respectively to obtain information regarding the structural properties and packing characteristics of the DPPC-DTX system and found that the presence of DTX promoted the ripple gel phase and increased the bilayer thickness in the liquid crystalline phase. The multilamellar organization of phospholipids generates a SAXD pattern with reflections distances relating as 1:1/2:1/3:1/4…, the largest first order reflection correlating with the interlamellar repeat distance (*d*) which includes the bilayer thickness and the thickness of the water layer between bilayers (Luzzati 1968; Tyler et al. 2015).

**Fig. 3:**
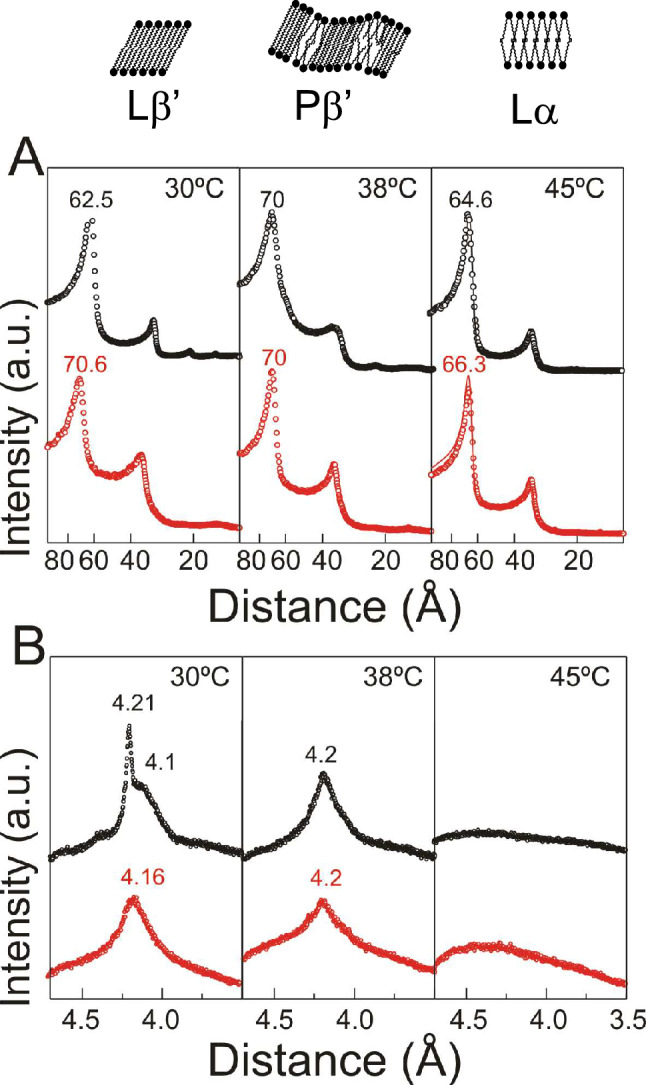
**A** Small angle X-ray diffraction (SAXD) profiles of pure DPPC (top) and DPPC containing DTX 0.08 molar fraction (bottom) at different temperatures. Solid lines at 45 °C represent the best fit to the experimental pattern using the GAP program. **B** Wide angle X-ray diffraction (WAXD) profiles of pure DPPC (top) and DPPC containing DTX 0.08 molar fraction (bottom) at different temperatures

Pure DPPC shows SAXD bilayers reflections with a d value of 62.5 Å at 30 °C, 70 Å at 38 °C, and 64.6 Å at 45 °C (Fig. 3A top). DPPC WAXS reflections are presented in Fig. 3B (top). At 30 °C the pattern shows a sharp reflection centred at 4.21 Å and a broad reflection at 4.10 Å which correlate with the quasi hexagonal lattice in which the acyl chains are tilted with respect to the bilayer normal forming one group of four closely spaced chains with two chains at a slightly larger separation characteristic of the L_β_’ gel phase (Tardieu et al. 1973). At 38 °C, a single reflection is present at 4.20 Å attributed to a lipid phase with hydrocarbon chains being oriented normal to the bilayer plane in a two-dimensional hexagonal lattice as defined for the P_β_’ gel phase (Lohner et al. 2001). At 45 °C, the WAXD pattern consists of a single broad diffuse reflection characteristic of the L_α_ liquid crystalline phase (Kriechbaum and Laggner 1996). All the aforementioned data reflect the DPPC successive transitions form the tilted gel phase to the ripple gel phase and then to the fluid phase and are in agreement with previous reports (Carion-Taravella et al. 2002; Berényi et al. 2013).

The SAXD patterns in the presence of DTX 0.08 molar fraction (Fig. 3A bottom) always show two reflections related as 1:1/2 indicating that the presence of the drug does not modify the lamellar structural assembly of DPPC. At 30 °C the *d* value for the mixture DPPC-DTX is considerably larger (70.6 Å) than that of the pure phospholipid (62.5 Å), and the WAXD pattern for the mixture only shows a single reflection at 4.16 Å. This WAXD reflection is broader than the corresponding reflection of conventional L_β_ phases formed in other phospholipid systems like phosphatidylethanolamine (Ortiz et al. 2008) revealing the tilt of the chains in the P_β_’ phase, and indicating that, at this temperature, the presence of DTX promotes the organization of the ripple gel phase. At 38 °C the system is still organized in the ripple phase. At 45 °C, the diffuse scattering reflection indicate that the mixture is organized in the liquid crystalline phase, however, the d value in the presence of DTX is larger (66.3 Å) than that of the pure phospholipid (64.6 Å).

**Fig. 4:**
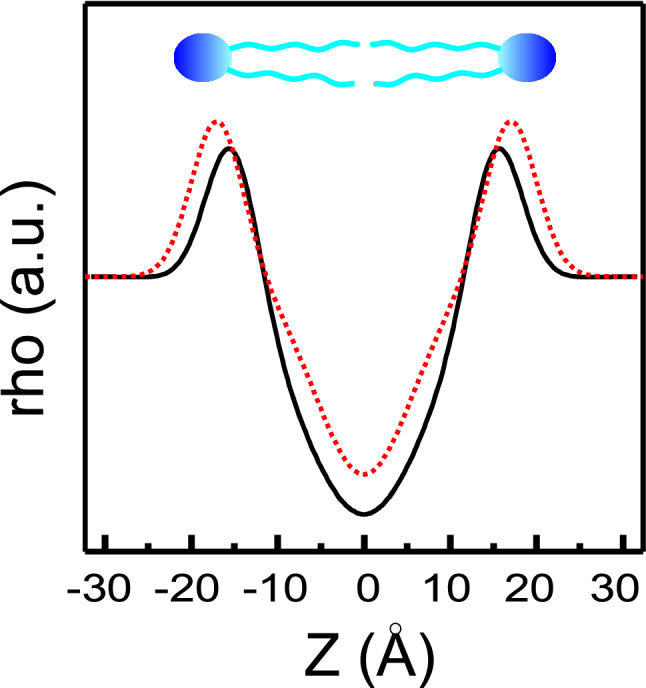
One-dimensional electron density profiles calculated from SAXD patterns of pure DPPC (solid line) and DPPC containing DTX 0.08 molar fraction (dotted line) at 45 °C, using the GAP program

In order to discern whether this difference in interlamellar repeat distance was due to a change in the bilayer thickness of to a change in the thickness of the water layer, background subtracted SAXD patterns for pure DPPC and DPPC containing DTX 0.08 molar fraction at 45 °C were examined using the GAP program (Fig. 3A solid lines). Figure 4 shows the correspondent one-dimensional electron density profiles along the bilayer normal calculated from the SAXD diffraction patterns. The profile for pure DPPC consists of a central region of comparatively low electron density values which correspond to the hydrocarbon chains of the phospholipid molecules; a region of relatively high electron density corresponding to the headgroups, which symmetrically border the hydrocarbon region; and an interstitial solvent-rich layer with electron density values intermediate between those of the first two regions. The bilayer is centred at the origin, so that the low electron density trough at 0 Å corresponds to the terminal methyl groups in the bilayer centre. For pure DPPC in the fluid phase we determined a bilayer thickness, *d*_B_, of 42.3 ± 0.2 Å, the presence of DTX increased the bilayer thickness to a value of 46.4 ± 0.2 Å (*p* = 0.003, *p* < 0.05, *n* = 3).

**Fig. 5:**
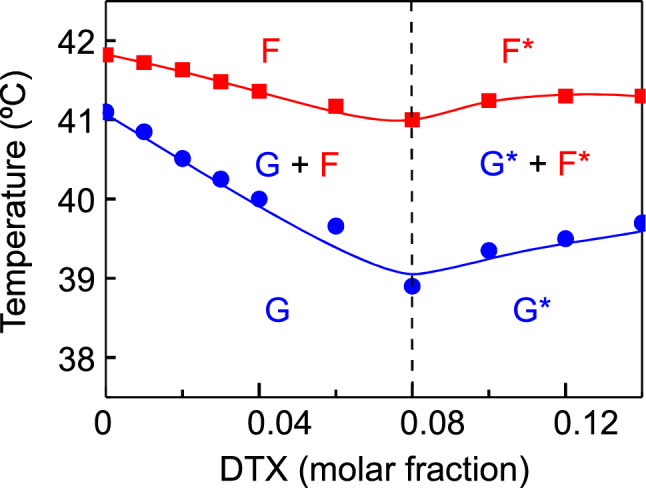
Partial phase diagrams for DPPC in DPPC/DTX mixtures. Circles and squares were obtained from the onset and completion temperatures of the main gel to liquid crystalline phase transition respectively. Circles, solidus line; squares, fluidus line. The phase designations are as follows: *G* gel phase; *F* liquid crystalline phase (fluid phase). The asterisk indicates that immiscible phase separation is present

A partial phase diagram for the DPPC component in mixtures with DTX has been composed using the phase transition temperatures from the DSC data and the structural characterization from the X-ray diffraction experiments, and is presented in Fig. 5. The solidus and fluidus lines display a near ideal behaviour, their temperatures decreasing with increasing concentrations of DTX, this decrease is less marked in the case of the fluidus line. Until a DTX concentration of 0.08 molar fraction, the system evolves from a gel phase (G) to a liquid crystalline phase (Fluid, F) through a coexistence region which is wider as more DTX is present. However, at concentrations of DTX higher than 0.08 molar fraction, the system behaves differently, both the solidus an fluidus lines keep horizontal, or even increase slightly their temperature, as more DTX is present in the system. The evidence that the main phase transition is not further perturbed when DTX is present at concentration higher than 0.08 molar fraction indicates the existence of immiscibility both in the gel and in the fluid phase. This phase separation may reveal an extensive aggregation of DTX inside the bilayer, but given the high molecular mass of DTX it probably reflects a limited solubility of DTX in the DPPC bilayer as shown previously for Paclitaxel (Balasubramanian and Straubinger 1994). Studies on the interaction of Paclitaxel with DPPC showed a qualitative similar behaviour with a maximum incorporation between 3% (Balasubramanian and Straubinger 1994; Belsito et al. 2005) and 5% (Bernsdorff et al. 1999; Ali et al. 2000; Zhao et al. 2004; Zhao et al. 2007). From a quantitative point of view DTX seems to better incorporate into DPPC bilayer, and this might enhance the accumulation of DTX in the target cell and contribute to the better efficacy that has been reported for the drug (Imran et al. 2020).

### Infrared Spectroscopy

**Fig. 6:**
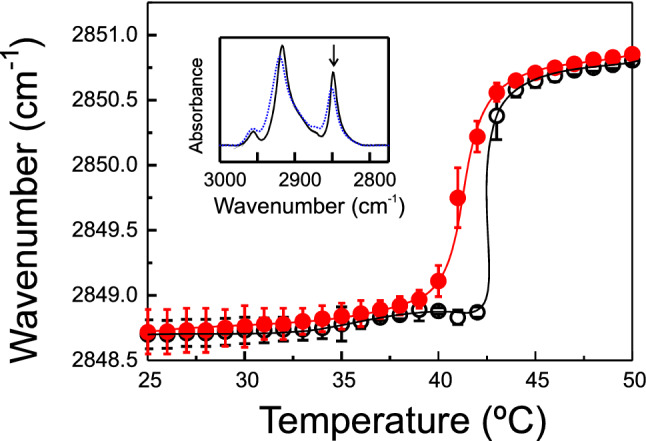
Temperature dependence of the maximum of the symmetric methylene stretching vibration band, sν (CH_2_), exhibited by pure DPPC (open circles) and DPPC containing DTX 0.08 molar fraction (filled circles). Symbols represent average and bars standard deviation of three different experiments. Inset shows the 2800–3000 cm^−1^ spectral region containing the absorption bands originating from the different carbon-hydrogen stretching vibrations of DPPC molecule at temperatures below (solid line) and above (dotted line) the main gel to liquid crystalline phase transition. The arrow points to the methylene symmetric stretching band

To investigate the effect of DTX on the hydrophobic and interfacial parts of the DPPC molecule, infrared spectroscopy was used and we found that the drug altered the lipid–water hydrogen bonding. Figure 6 shows the temperature dependence of the frequency at the absorbance maximum of the symmetric methylene stretching vibration band, sν (CH_2_), of the infrared spectra of pure DPPC and that of the system containing DTX 0.08 molar fraction. The inset of Fig. 6 shows the 3000–2800 cm^−1^ spectral region containing the absorption bands originating from the different carbon-hydrogen stretching vibrations of DPPC molecule at temperatures below and above the main gel to liquid crystalline phase transition, the arrow points to the methylene symmetric stretching band. This band is of special importance because, being free of overlapping contributions from other groups, is susceptible to changes in the mobility and conformational disorder of the phospholipid acyl chains (Lewis and McElhaney 2006). For pure DPPC in the gel tilted phase the absorption maximum of the band is detected close to 2848.7 cm^−1^, this value increases slightly to 2849 cm^−1^ in the rippled gel phase, and then raises abruptly during the main phase transition reaching wavenumbers close to 2851 cm^−1^ at 50 °C. This rise in frequency, which appears with a broadening of the absorption band (see inset of Fig. 5), is indicative of the gel to liquid crystalline phase transition of hydrated phospholipids and come from the expanded conformational disorganization in the phospholipid acyl chains that takes place at the phase transition (increase in the *gauche*/*trans* conformer ratio) (Mantsch and McElhaney 1991). In the system containing DTX, the drug makes the small increase occurring at the pretransition to vanish and generates a broadening of the transition which is relocated to lower temperature values in consonance with the DSC experiments presented above. The increase in wavenumber taking place at the main phase transition both in pure DPPC and in the presence of DTX is identical (approx. 2 cm^−1^) and this agrees with the evidence from DSC that the enthalpy change for the phase transition is not altered in the presence of the drug.

It has been reported that incorporation of 10 mol% Paclitaxel produced a very small increase of the wavenumber of the maximum of this band both in the gel and the liquid crystalline phase of DPPC, and contrary to that, the presence of Paclitaxel in dimirystoylphosphatidylserine systems induced a slight decrease of the wavenumber in both phases (Bernsdorff et al. 1999). Studies on the effect of Paclitaxel on the fluidity of phospholipid bilayers using steady-state anisotropy produced also differing results, while Paclitaxel produced a fluidizing effect in the gel phase and had no effect in the liquid crystalline phase of DPPC (Balasubramanian and Straubinger 1994), a slight rigidification effect was reported in unsaturated phosphatidylcholines (Bernsdorff et al. 1999). In our case for DTX, as observed in Fig. 6, the change in *gauche*/*trans* conformer ratio both in the gel and the liquid crystalline phase are not substantial, the slight effects of the drug being within the experimental error.

**Fig. 7:**
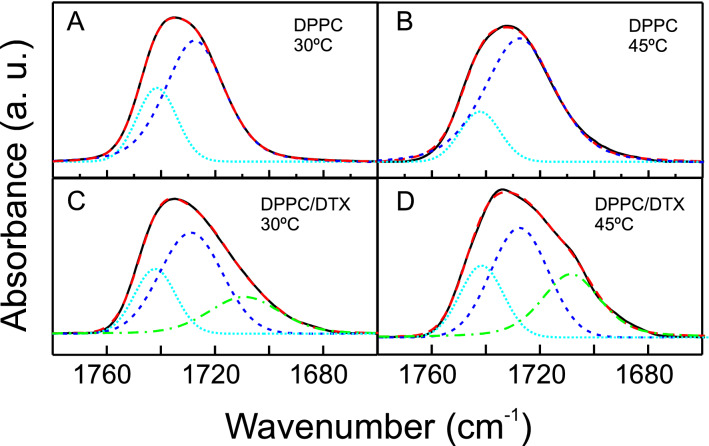
Representative FTIR spectra illustrating the components of the ester carbonyl stretching band, ν(C = O), exhibited by DPPC bilayers at temperatures belove (**A**) and above (**B**) the lipid gel/liquid-crystalline phase transition; and by DPPC containing DTX 0.08 molar fraction at temperatures below (**C**) and above (**D**) the lipid gel/liquid-crystalline phase transition. The absorbance spectra shown were acquired at the temperatures indicated with the different lines representing: solid line, observed baseline corrected spectra; large dashed line, fitted spectra; dotted line, estimates of the component band appearing at 1742 cm^−1^; short dashed line, estimates of the component band appearing at 1728 cm^−1^; and dashed-dotted line, estimates of the component band appearing at 1711 cm^−1^, as determined by curve fitting

Interestingly, the presence of DTX originates striking changes in the ester carbonyl stretching band, ν(C = O), of the infrared spectra of DPPC which appears near 1730 cm^−1^. The ν(C = O) region of the spectra of phospholipids incorporates clues about phospholipid interfacial hydration and hydrogen bonding interplay, and hence supply specifics about the intermolecular interactions that take place in this region. As seen in Fig. 7AB, the ν(C = O) band of DPPC is a rather broad one over 1760–1680 cm^−1^, in accordance with previous reports (Mantsch and McElhaney 1991). It is accepted that this band is an addition of two subcomponents bands, one located near 1742 cm^−1^ reflecting a subpopulation of non-hydrogen bonded carbonyl groups and another one located near 1728 cm^−1^ reflecting a subpopulation of hydrogen bonded carbonyl groups (Lewis et al. 1994).

Pure DPPC exhibits band maxima near 1733.6 cm^−1^ in the gel phase (Fig. 7A) and near 1731.6 cm^−1^ in the liquid crystalline phase (Fig. 7B) in agreement with previous report (Chicano et al. 2001). This variation in the frequency maxima to lower values reflects the increment in band intensity of the subjacent subcomponent band near 1728 cm^−1^ which is associated to a greater extent of hydrogen bonded carbonyl groups originating from the increase in the hydration of the polar-nonpolar interface induced during the phase transition to the liquid crystalline state (Zhang et al. 1997). In the presence of DTX the maxima of the carbonyl band appear at higher frequencies than that of the pure DPPC, in the gel phase (Fig. 7C) the maximum is located near 1735.1 cm^−1^ and in the liquid crystalline phase (Fig. 7D) is located near 1734.3 cm^−1^. The displacement of the carbonyl band maxima to higher wavelength suggests that there would be a major contribution of the subpopulation of non-hydrogen bonded carbonyl group and hence a higher degree of dehydration of the lipid polar-nonpolar interface in the presence of the drug.

To assess the possibility that the presence of DTX would produce a less hydrogen bonded interfacial region of DPPC bilayers, the different ester carbonyl stretching spectra were submitted to curve fitting through simulation by a Gaussian–Lorenztian function, and the results are presented also in Fig. 7. In agreement with previous reports (Lewis et al. 1994, 1996), the spectra corresponding to pure DPPC were best fitted to two component: a narrower component at 1741 cm^−1^ and a broader one at 1728 cm^−1^, as commented above. In the system containing DTX no good fit to two components could be obtained. It has been reported that DTX exhibits carbonyl absorption maximum near 1711 cm^−1^ (Fang et al. 2015; Albano et al. 2019; Jose et al. 2019). Even though the presence of DTX in our samples is low (0.08 molar fraction) the number of carbonyl groups in the DTX molecule makes their contribution significant. We found that the band profile could be fitted to a composite of three components with maxima near 1742, 1728 and 1711 cm^−1^, and we assigned the lower wavelength carbonyl component to the absorption of DTX carbonyl. The simulated components were analysed and their relative areas were calculated.

Table 1 displays an overview of the temperature dependent variation in the band contribution of the two ester carbonyl stretching components of DPPC displayed by pure DPPC and DPPC containing DTX, omitting the contribution of the DTX carbonyl component at 1711 cm^−1^. In the case of pure DPPC, in close accordance with previous results (Mannock et al. 2008; Silva et al. 2011), we found that the contribution of the non-hydrogen bonded carbonyl component is around 25% and 19% in the gel phase and the liquid crystalline phase respectively. The presence of DTX is associated with an increase in the relative area of the component band located near 1742 cm^−1^ corresponding to non-hydrogen bonded carbonyls groups. In the gel phase the contribution of this component increases from 25% for pure DPPC to 29.5% in the presence of DTX, however, in the liquid crystalline phase this increase is much larger changing from 19% for pure DPPC to 33.3% in the presence of drug. These findings are especially noteworthy because they indicate that the presence of DTX generate not only a change in the hydrogen-bonding interactions in the interfacial region of the bilayer, but also a dehydration effect which is more noticeable in the liquid crystalline phase.

**Table 1:** Characterization of the components of the DPPC ester carbonyl stretching band in systems containing pure DPPC and DPPC/DTX 0.08 molar fraction

Peak maximum (cm^−1^)	Peak area	(% total)
	Gel phase (30 °C)	Fluid phase (45 °C)
*DPPC*		
1742	25.08 ± 2.47	19.08 ± 6.33
1728	74.78 ± 1.81	80.91 ± 6.34
*DPPC + DTX*		
1742	29.55 ± 0.41	33.26 ± 2.33
1728	70.45 ± 0.41	66.74 ± 2.33

Values represent average ± SD of three different experiments. Differences between temperatures and presence of DTX were significant (*p* < 0.05)

### Molecular Dynamics

**Fig. 8:**
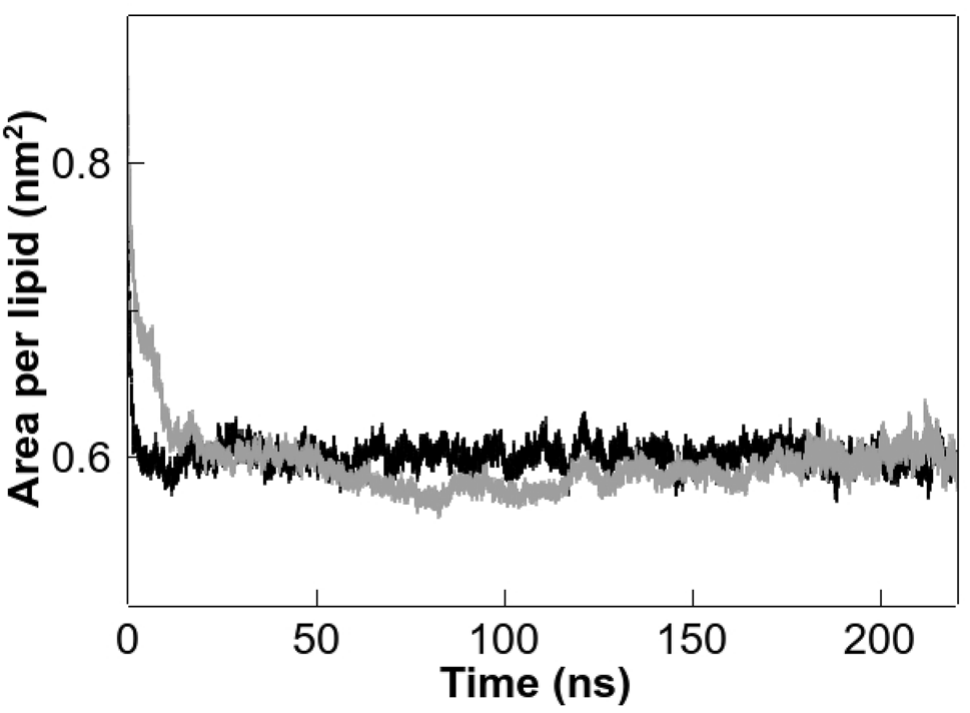
Area per lipid vs. simulated time for pure DPPC (black line) and DPPC containing DTX (grey line). Last 60 ns were used for all MD analysis

Computer simulation, such as MD, has proven to be an important contribution to biophysical research on the physicochemical properties of lipid membranes, as it provides atomic detail of the simulated system (Friedman et al. 2018). The area per lipid at the membrane aqueous interface is frequently used as a property of the lipid bilayer for validating MD simulations and as a proof of convergence (Venable et al. 2019)⁠. Figure 8 shows the progression of the area per lipid of the simulation runs, where it can be observed that the area per lipid reach convergence and keeps constant in the time range used for all the analysis (last 60 ns). The area per lipid is calculated from the lateral dimensions of the simulation box (the area of the x y plane) divided by the number of lipids in each leaflet. In our simulation the area per lipid of pure DPPC bilayer above the phase transition (50 °C) was calculated to be 60 ± 0.7 Å^2^, this value is among reported data for this phospholipid membrane (Schindler and Seelig 1975; Nagle 1993; Kučerka et al. 2008)⁠. The presence of DTX in the DPPC membrane did not significantly alter the area per lipid [60 ± 1.0 Å^2^, *p* = 0.59 (*p* > 0.05)].

Membrane thickness has been calculated from the distance between the averaged z-positions of the phosphorus atoms of opposing leaflets. For a pure DPPC membrane, we obtained a distance of 39.5 ± 1.3 Å, which is well among previously reported data (Nagle and Tristan-Nagle 2000; Kučerka et al. 2011). In the presence of DTX we found a significant increase in the membrane thickness to 46.7 ± 0.9 Å, *p* = 0.0012 (*p* < 0.05). These results are in line with the calculated d_B_ values from our diffraction X-ray experiments (Fig. 4). It has been proposed that integral membrane proteins have hydrophobic parts that are in contact with the phospholipid acyl chains and are essential for balanced integration of the protein into the bilayer (Killian 1998). Hydrophobic mismatch takes place when the hydrophobic length of the integral protein does not match the hydrophobic thickness of the membrane, this alteration of the membrane properties could be responsible for lipid-dependent protein function as it could produce some alteration in the structure of the protein (Cybulski and de Mendoza 2011). Taking into account that integral membrane proteins are responsible for important cellular processes, the increase in the bilayer thickness exerted by DTX might be important when considering the hydrophobic mismatch and may have a potential in the mechanism of action of some of the toxic effect of the drug.

The criteria adopted to assume hydrogen bonds formation was that the distance between the water hydrogen and the DPPC oxygen be shorter than or equal to 3.5 Å and the H-bond angle less than or equal to 30°. For pure DPPC, we obtained a result of 1.48 ± 0.02 hydrogen bonds from water to the carbonyl oxygen atoms of DPPC per phospholipid molecule, which is within reported values which range from 1.2 to 2.18 (Pasenkiewicz-Gierula et al. 1997; Leekumjorn and Sum 2006; Elola and Rodriguez 2018). The presence of DTX in the membrane decreases the hydrogen bonds number to 1.36 ± 0.01, *p* = 0.0013 (*p* < 0.05), which is consistent with the dehydration observed in the carbonyl group by IR spectroscopy (Table 1).

We found a value of 0.47 ± 0.03 hydrogen bonds from water to carbonyl oxygen atoms of DTX per phospholipid molecule. The presence of an additional low frequency phospholipid carbonyl component has been reported for saturated 1,2-diacylphosphatidylethanolamines (Lewis and McElhaney 1993), acyl–alkyl analogues of phosphatidylcholines (Lewis et al. 1994, 1996), and also for mixtures of DPPC and highly lipophilic compounds (Jiménez et al. 2002). However, in our case we found that the formation of hydrogen bonds between the carbonyl of DPPC and other groups different from water (for example with DTX) were very minor. The latter suggest that the lower wavelength carbonyl component indeed corresponded to the carbonyl of DTX and suggest that the establishment of hydrogen bonds between DTX carbonyls and water contributes to the decreased number of bonds between DPPC carbonyls and water, as found in our FTIR measurements.

**Fig. 9:**
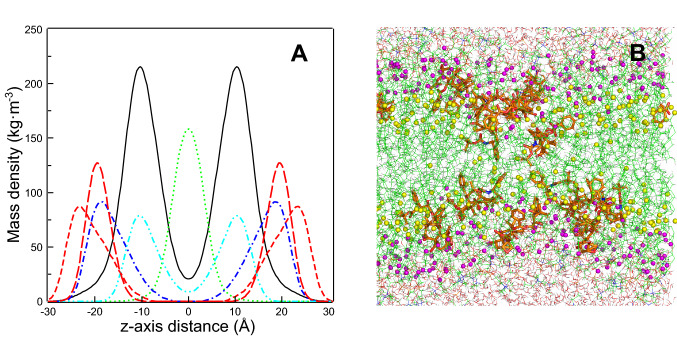
**A** Mass density profiles along the z-axis of the simulation box at 50 °C of the molecular simulations. DTX molecule in solid line, phosphorus atom of DPPC in dashed lines (large dashed for pure DPPC and short dashed for DPPC + DTX), C16 atoms (methyl terminals of the hydrocarbon chains) of DPPC with DTX in dotted line, C1 atoms (carbonyl groups) of DPPC with DTX in dash one dot line, and C8 atoms of DPPC with DTX in dash two dots line. **B** Final snapshot of the simulation box at 50 °C of DPPC + DTX. Water molecules are shown in lines, DTX in sticks, DPPC in lines, C8 atoms of the DPPC hydrocarbon chains in light spheres, and C1 atoms of the DPPC hydrocarbon chains (carbonyl groups) in dark spheres

The mass density of the simulated membranes at 50 °C is shown in Fig. 9A. Some important positions of the DPPC molecule along z-axis have been included: the terminal methyl groups to show the center of the membrane (C16 atoms), the center of the hydrocarbon chains of DPPC (C8 atoms), the carbonyl groups (C1 atoms), and the polar head region (P atoms). It can be seen that DTX molecules are located in the hydrophobic core of the membrane, being centered at about the C8 atoms of the hydrocarbon chains of DPPC, the center of the hydrophobic core of a monolayer. However, DTX band overlaps well with the carbonyl groups and even reaches the P atoms band in the polar head group region.

Figure 9B shows a snapshot of the simulation box at 50 °C of DPPC/DTX mixture where the location of DTX in the bilayer can be observed. This is the expected location for DTX considering the experimental results commented above, the location near C8 atom allows DTX to perturb the gel to liquid crystalline phase transition and the proximity to the carbonyl region do DPPC enable the drug to interfere with the hydrogen bonding pattern of the interfacial region of the bilayer.

**Fig. 10:**
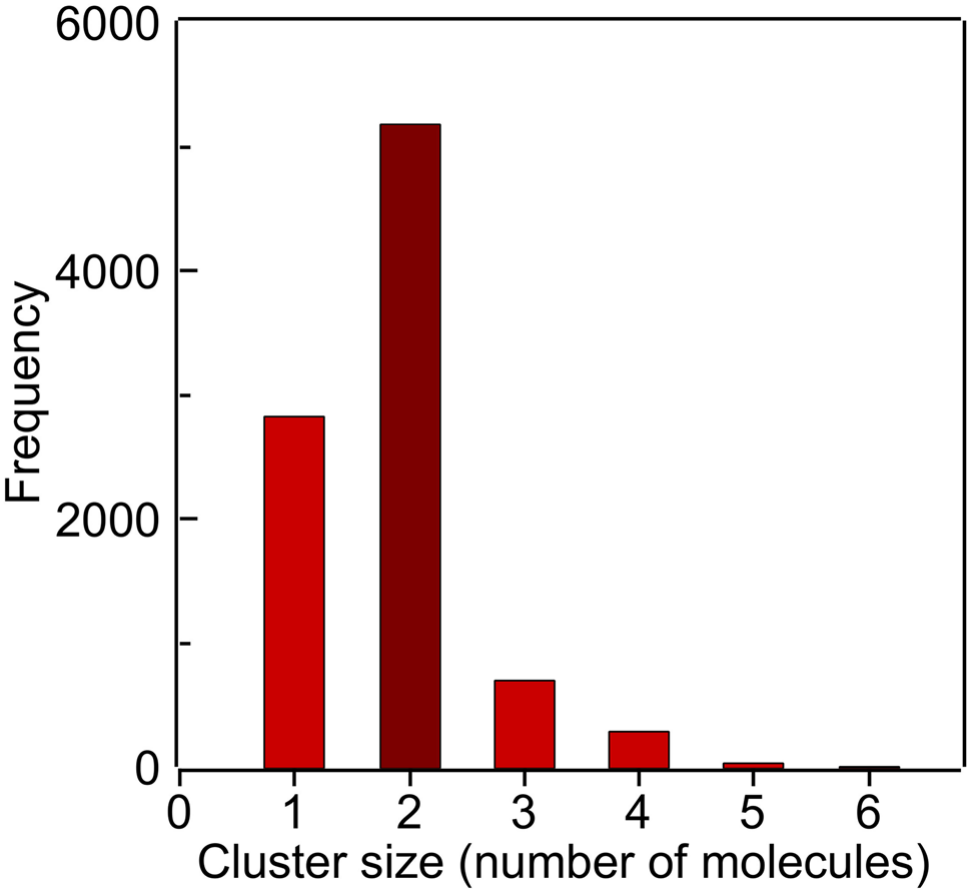
Frequency of formation of different size DTX clusters in the bilayer within a cutoff distance of 3 Å during the simulation

The tendency of DTX to aggregate in the membrane has been determined by calculating the frequency of formation of different size of DTX clusters within a cutoff distance of 3 Å (Fig. 10). Most of the DTX molecules are found in the DPPC membrane in clusters of two molecules, although bigger cluster sizes of 3 and 4 molecules can also be observed, thus it can be concluded that most of DTX molecules are forming aggregates in the membrane. The presence of these different populations of DTX in the fluid bilayer may explain the appearance of two different domains which are responsible for the two slightly separate transitions which conformed the thermogram in the presence of the drug (Fig. 2D); the presence of clusters may also justify the modest decrease in temperature of the fluidus line in comparison with the solidus line when the concentration of DTX is increased (Fig. 5).

## Conclusions

Molecular interactions between DTX and DPPC were investigated with biomimetic bilayer membranes using a combined experimental and computational approach. DSC studies have shown that DTX is miscible with the phospholipid bilayer only to a limited extent, perturbing the pretransition and the main gel to liquid crystalline phase transition, broadening and shifting the transition temperature to lower values, and giving rise to immiscible phase separation when the presence of the drug is higher than 0.08 molar fraction. Analysis using X-Ray Diffraction revealed that the drug promotes the formation of the ripple phase in the gel state and increased the bilayer thickness in the liquid crystalline phase. FTIR experiments illustrated how the decrease in the transition temperature could be followed through the change in gauche conformers of the methylene chains of the phospholipid, and established the alteration in the hydrogen-bonding interactions produced by DTX on the interfacial region of the bilayer conducing to a dehydration of the region. The experimental conclusions supported the results of Molecular Dynamics were an increase in the bilayer thickness and a dehydration effect were also postulated. Simulation experiments located the anticancer molecule forming small clusters in the region of the carbon 8 of the phospholipid acyl chain palisade overlapping with the interfacial carbonyl region in agreement with the effect observed by experimental techniques. We believe that the observed interactions between DTX and DPPC generate physical disturbances which could alter membrane function, and may help to discern the mechanism of action of the increasing list of biological action of DTX.

## Data Availability

Data sharing not applicable to this article as no datasets were generated or analysed during the current study.
